# Low-frequency HIV-1 drug resistance mutations in antiretroviral naïve individuals in Botswana

**DOI:** 10.1097/MD.0000000000029577

**Published:** 2022-07-15

**Authors:** Dorcas Maruapula, Kaelo K. Seatla, Olorato Morerinyane, Kesaobaka Molebatsi, Jennifer Giandhari, Tulio de Oliveira, Rosemary M. Musonda, Melvin Leteane, Sununguko W Mpoloka, Christopher F. Rowley, Sikhulile Moyo, Simani Gaseitsiwe

**Affiliations:** a Botswana Harvard AIDS Institute Partnership, Gaborone, Botswana; b Department of Biological Sciences, University of Botswana, Gaborone, Botswana; c School of Allied Health Professions, University of Botswana, Gaborone, Botswana; d Department of Statistics, University of Botswana, Gaborone, Botswana; e KwaZulu-Natal Research Innovation and Sequencing Platform (KRISP), School of Laboratory Medicine and Medical Sciences, College of Health Sciences, University of KwaZulu-Natal, Durban, South Africa; f Department of Immunology and Infectious Diseases, Harvard T.H. Chan School of Public Health, Boston, MA.

**Keywords:** antiretroviral naïve, HIV drug resistance, next-generation sequencing, low-frequency variants

## Abstract

**Background:**

Individuals living with human immunodeficiency virus (HIV) who experience virological failure (VF) after combination antiretroviral therapy (cART) initiation may have had low-frequency drug resistance mutations (DRMs) at cART initiation. There are no data on low-frequency DRMs among cART-naïve HIV-positive individuals in Botswana.

**Methods:**

We evaluated the prevalence of low-frequency DRMs among cART-naïve individuals previously sequenced using Sanger sequencing. The generated pol amplicons were sequenced by next-generation sequencing.

**Results:**

We observed low-frequency DRMs (detected at <20% in 33/103 (32%) of the successfully sequenced individuals, of whom four also had mutations detected at >20%. K65R was the most common low-frequency DRM detected in 8 individuals. Eighty-two of the 103 individuals had follow-up viral load data while on cART. Twenty-seven of the 82 individuals harbored low-frequency DRMs. Only 12 of 82 individuals experienced VF. The following low-frequency DRMs were observed in four individuals experiencing VF: K65R, K103N, V108I, and Y188C. No statistically significant difference was observed in the prevalence of low-frequency DRMs between individuals experiencing VF (4/12) and those not experiencing VF (23/70) (*P* = .97). However, individuals with non-nucleoside reverse transcriptase inhibitors-associated low-frequency DRMs were 2.68 times more likely to experience VF (odds ratio, 2.68; 95% confidential interval, 0.4–13.9) compared with those without (*P* = .22).

**Conclusion:**

Next-generation sequencing was able to detect low-frequency DRMs in this cohort in Botswana, but these DRMs did not contribute significantly to VF.

## 1. Introduction

Treatment of human immunodeficiency virus (HIV) with combination antiretroviral therapy (cART) is life-long and has high success in suppressing HIV-1 replication.^[1,2]^ However, cART success can be negatively impacted by the emergence of HIV drug resistance mutations (DRMs), leading to virological failure (VF).^[3–6]^ Dolutegravir (DTG)-based regimens have been adopted as the preferred first-line treatment for HIV, replacing non-nucleoside reverse transcriptase (RT) inhibitor (NNRTI)-based regimens.^[7,8]^ However, concerns have emerged about the safety of DTG^[9]^ and its reduced efficacy in patients with DRMs in RT.^[10]^ Furthermore, some countries are opting to continue using efavirenz (EFV)-based first-line cART, especially in patients receiving tuberculosis (TB) treatment due to the risk of increased DTG metabolism, leading to subtherapeutic concentrations.^[11,12]^ With expanded access to cART, there is increased potential for the development and transmission of drug-resistant HIV variants, leading to treatment failure.^[13]^ Population-based Sanger sequencing is widely used in HIV drug resistance testing.^[14]^ However, population-based Sanger sequencing only detects the most dominant viral variants (>20% of the viral quasispecies) and is unable to detect low-frequency DRMs (minority variants).^[15,16]^ Next-generation sequencing (NGS) allows for the effective detection of low-frequency DRMs as low as 1% of the viral population^[17–23]^ and can be cost-effective by using the pooling strategy, which pools index samples into a single library.^[24–26]^

Previous studies^[27–39]^ have reported that individuals initiating cART with preexisting HIV low-frequency DRMs have a higher likelihood of VF, particularly among those initiating NNRTIs. It has also been reported that individuals with preexisting low-frequency DRMs at baseline have the same DRMs at the time of VF.^[40,41]^ However, other studies have found no association between baseline low-frequency DRMs and VF on cART.^[40,42,43]^

Conflicting results on the clinical importance of low-frequency DRMs indicate the need for further investigations on the clinical impact of low-frequency DRMs in different settings.

We sought to determine the prevalence of low-frequency DRMs among cART-naïve HIV-positive individuals in Botswana and to assess the impact of baseline pretreatment low-frequency DRMs on VF outcomes once the participants initiated cART.

## 2. Materials and Methods

### 2.1. Study design and study population

This was a retrospective longitudinal study aimed at determining the prevalence and impact of low-frequency HIV DRMs in baseline samples of antiretroviral naïve individuals in Botswana. The amplicons used in this study were obtained from a previous Botswana Harvard AIDS Partnership study (BHP063- with protocol title: A novel strategy for HIV drug resistance monitoring in developing countries).^[44]^ Participants were recruited for the study from antenatal clinics and Infectious Disease Care Clinics in 3 different locations in Botswana: Gaborone, Molepolole, and Mochudi. BHP063 enrolled 443 participants between April 2012 and April 2015 and were included in the primary study analysis.^[44]^ An additional 88 participants were enrolled between May 2015 and December 2015, resulting in a total of 531. Before 2016, the standard first-line of ART initiation used included the combination of EFV, tenofovir disoproxil fumarate (TDF), and emtricitabine (FTC) (ATRIPLA) (Gilead Sciences), and there were no patients on DTG-based ART before then. As of June 2016, Botswana adopted DTG-based ART (DTG-TDF-FTC) for all HIV-infected individuals regardless of CD4+ T-cell count or pregnancy status. Most of these participants were probably infected through heterosexuals given their age at the time of enrollment, but mother-to-child transmission cannot be ruled out. Genotyping results for participants in the main cohort who previously developed resistance mutations were communicated to clinicians, and patients were assigned treatment based on baseline mutation status. This would impact their treatment. Of the 531 enrolled in BHP063, 108 (20.3%) participants with available stored HIV-1 RT/PR amplicons and available Sanger sequencing data were included in the present study. Four hundred and twenty-three (423) participants without amplicons were excluded. The characteristics of the included and excluded individuals were compared (Table S1, Supplemental Digital Content, http://links.lww.com/MD/G927). Follow-up clinical, virological, and demographic data for individuals with available amplicons were extracted from the electronic laboratory information system-IPMS (Integrated patient management system). We defined virological suppression as viral load (VL) <400 copies/mL and VF as VL of ≥400 copies/mL at 6 months after initiation of cART as per the Botswana Ministry of Health and Wellness guidelines.

### 2.2. Ribonucleic acid extraction, polymerase chain reaction amplification, and Sanger sequencing

The HIV-1 *pol* (RT/PR) amplicons were initially generated in a previous study.^[44]^ Briefly, ribonucleic acid (RNA) was extracted from 400 ul plasma samples using an EZ1 Virus Mini Kit v2.0 (Qiagen, Valencia, CA) on an EZ1 Advanced XL (Qiagen) automated instrument. The RT and protease (PR) regions of the HIV-1 pol gene were amplified, and polymerase chain reaction (PCR) products were purified using a QIAquick PCR purification kit (Qiagen, Hilden, Germany), according to the manufacturer’s instructions. DNA sequencing of PCR products was performed using BigDye Terminator chemistry on an ABI 3130XL genetic analyzer (Thermo Fisher Scientific, Carlsbad, CA), as previously described.^[44]^ The residual amplicons were stored at −20°C.

### 2.3. Next-generation sequencing and drug resistance analysis

NGS was conducted at KwaZulu-Natal Research Innovation and Sequencing Platform, Durban, South Africa, and Inqaba Biotechnical Industries, Pretoria, South Africa, using the Illumina MiSeq platform (Illumina, San Diego, CA). Briefly, PCR product concentrations were determined using a Qubit 3.0 fluorometer (Thermo Fisher, Malaysia). Paired-end libraries were generated using the Nextera-XT DNA library preparation kit and Nextera Index kit (Illumina, San Diego, CA), according to the manufacturer’s instructions. Sequencing libraries were purified using Agencourt AMPure XP beads, and quantified, and barcoded libraries were pooled for sequencing on an Illumina MiSeq platform. The generated raw reads (FastQ files) were assembled into contigs using online genome detection tools.^[45]^ NGS sequences were uploaded to the online variant caller polymorphism analysis sequencing (PASeq).^[46]^ HyDRA was also used to confirm the minor variants.^[23]^ Any variants not called by either caller were assumed to have a 0% allele frequency. Low-frequency DRMs were detected at >1% using Geneious software v8.1.9 (Biomatters Ltd, Auckland, New Zealand).^[47]^

### 2.4. Statistical analysis

HIV drug resistance was determined based on NGS with detection thresholds of 1%, 5%, 10%, and 20%. Data are presented as medians and interquartile ranges. The demographic characteristics (age and sex) and clinical characteristics (baseline CD4+ T-cell count and baseline VL) of individuals with and without pretreatment low-frequency DRMs were compared using the Wilcoxon rank-sum test and Fisher’s exact test (for continuous and categorical variables). We excluded individuals without follow-up VL data from the analysis to determine the impact of pretreatment low-frequency DRMs on VF. We further excluded pretreatment DRMs detected at ≥20% of mutations. We used a univariate exact logistic regression model^[48,49]^ to assess the association between pretreatment low-frequency DRMs and VF. Odds ratios (OR) were used to describe the association between low-frequency DRMs and VF. We used R version 4.0.3 for statistical analysis. Differences were considered statistically significant at *P* < .05.

### 2.5. Ethics

BHP063 study was approved by the Research Ethics Committee of the Ministry of Health and Welfare (HRDC # 00638). In addition, ethical approval was obtained from the Institutional Review Board of the University of Botswana (Reference number: HPDME 13/18/1 Vol 833). Participants provided informed consent for the reuse and storage of their samples for further research.

## 3. Results

### 3.1. Participants characteristics

**Figure 1.**
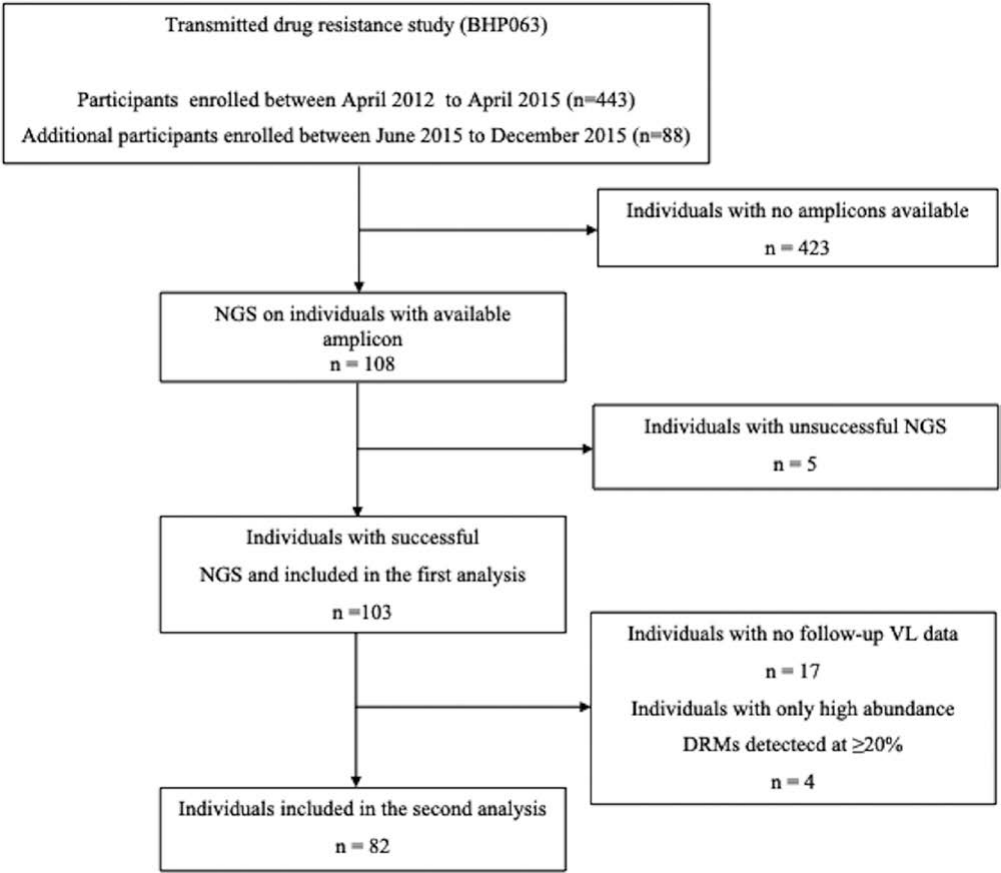
Flow chart showing individuals included in the analysis. The first analysis focused on determining the prevalence of low-frequency drug resistance mutations. The second analysis only used participants with follow-up viral load data to assess the impact of low-frequency drug resistance mutations on virological outcomes. DRM, drug resistance mutations; NGS, next-generation sequencing.

A total of 103 out of 108 samples were successfully sequenced using Illumina MiSeq NGS (Fig. 1) to a mean depth of 20534 reads (min–max: 1849–159407). At baseline, the median VL was 4.1 log_10_ copies/mL and the median CD4+ T-cell count was 365 cells/mm^3^. Table 1 summarizes the demographic and clinical characteristics of the participants.

**Table 1:** Baseline characteristics of participants included in the study.

Characteristics	Participants (n = 108)
Age in years, median (IQR)	27.0 (24–31)
Female, n (%)	107 (99.1%)
Male, n (%)	1 (0.9)
VL (log_10_ copies/mL), median (IQR)	4.1 (3.5–4.6)
CD4+ T-cell count (cells/mm^3^), median (IQR)	365 (225–497)

IQR = interquartile ranges: 25th percentile and 75th percentile; VL, viral load.

Of the 108 samples with available amplicon, 26 individuals had to be excluded from the second analysis of the study because of either unsuccessful NGS, presence of baseline pretreatment DRMs only, or no follow-up VL data (Fig. 1). Demographic characteristics of individuals included (n = 108) and excluded (n = 423) from the main cohort were summarized, and there was no significant difference in terms of age and VL between the individuals included and those excluded, but a significant difference was observed in sex and CD4 (Table S1, Supplemental Digital Content, http://links.lww.com/MD/G927).

### 3.2. HIV drug resistance mutations detected in protease and reverse transcriptase

Eight (7.8%) of the 108 individuals with successful NGS sequencing had at least 1 DRM detected at >20% frequency. Seven of the 8 sequences revealed single-class resistance (1 nucleoside RT inhibitor (NRTI), two NNRTI, and four PR inhibitors (PIs), whereas the other harbored both NRTI and NNRTI resistance. The most common mutation was K103N, which was detected in 3/8 sequences. PASeq and HyDRA detected all DRMs (>20% frequency), as determined by Sanger sequencing. The 2 analysis pipelines (PASeq and HyDRA) had a good agreement above 20% and 5% thresholds but gave highly discrepant results around a 1% threshold (Table S2, Supplemental Digital Content, http://links.lww.com/MD/G927). The variants called by PASeq were used to represent the NGS results for subsequent analyses.

**Figure 2.**
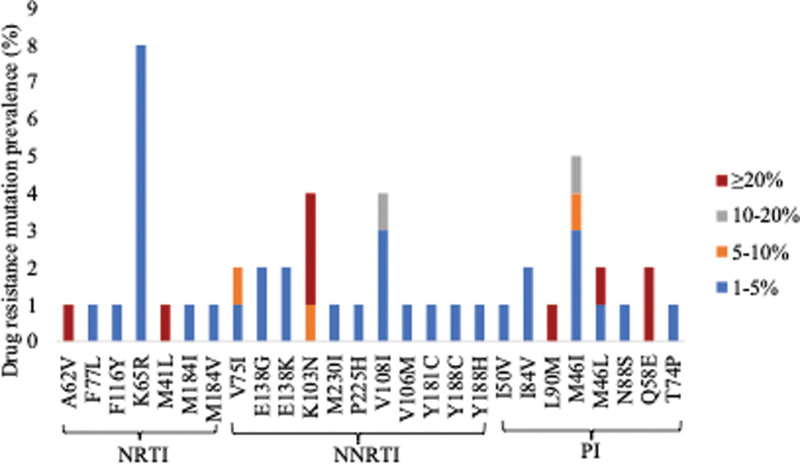
Baseline HIV drug resistance mutations detected at different thresholds. Mutations detected at 1 to <20% represent low-frequency mutations detected by next-generation sequencing only. Mutations detected at ≥20% represent mutations detected by both Sanger sequencing and next-generation sequencing. NRTI, Nucleoside reverse-transcriptase inhibitor; NNRTI, non-nucleoside reverse transcriptase inhibitor; PI, Protease inhibitor.

Thirty-three individuals (32.0%) had at least 1 low-frequency DRMs (1%–20% frequency). Four of the 33 participants also had DRMs at a ≥20% threshold (Fig. 2 and Table 2). Among the 33 individuals, NRTI-associated low-frequency DRMs were detected in 12 individuals. In addition, 2 individuals harbored both NRTI-and NNRTI-associated low-frequency DRMs. The most common NRTI-associated low-frequency DRM was K65R, occurring in 8 individuals with frequencies between 1% and 2.96%. Other NRTI-associated low-frequency DRMs identified were V75I, F77L, F116Y, M184I, and M184V at frequencies from 2.43% to 4.06%, respectively. NNRTI-associated low-frequency DRMs were found in 8 individuals. In addition, 2 individuals had both NNRTI-and PI-associated low-frequency DRMs. The most common NNRTI-associated low-frequency DRM was V108I, detected in 3 individuals with frequencies between 1.15% and 11.25%. Other NNRTI-associated low-frequency DRMs identified were K103N, V106M, E138G, E138K, Y181C, Y188C, Y188H, P225H, and M230I at frequencies of 1.01% and 7.7%, respectively. PI-associated low-frequency DRMs were found in 9 individuals, with the most common being M46I found in 5 individuals. Other PI-associated low-frequency DRMs identified were M46L, I50V, T74P, I84V, and N88S at frequencies of 1.13% and 3.38%, respectively.

**Table 2:** Drug class mutations observed by NGS at different mutation thresholds.

Mutations detected	Detection threshold				
	1%–2%	>2%–5%	>5%–10%	>10%–<20%	≥20%
NRTI-associated					
M41L	0	0	0	0	1
A62V	0	0	0	0	1
K65R	6	2	0	0	0
V75I	0	1	1	0	0
F77L	0	1	0	0	0
F116Y	0	1	0	0	0
M184I	0	1	0	0	0
M184V	0	1	0	0	0
Total	6	7	1	0	2
NNRTI-associated					
K103N	0	0	1	0	2
V106M	1	0	0	0	0
V108I	1	1	0	1	0
E138G	1	1	0	0	0
E138K	1	1	0	0	0
Y181C	1	0	0	0	0
Y188C	0	1	0	0	0
Y188H	1	0	0	0	0
P225H	1	0	0	0	0
M230I	1	0	0	0	0
Total	8	4	1	1	2
PI-associated					
M46I	2	2	0	1	0
M46L	0	1	0	0	0
I50V	1	0	0	0	0
T74P	1	0	0	0	0
I84V	1	1	0	0	0
N88S	0	1	0	0	0
Q58E	0	0	0	0	2
Total	5	5	1	1	2

NGS = next-generation sequencing; NRTI = nucleoside reverse-transcriptase inhibitor; NNRTI = non-nucleoside reverse transcriptase inhibitor; PI = Protease inhibitor. Mutations detected at >20% were also detected by Sanger sequencing.

**Figure 3.**
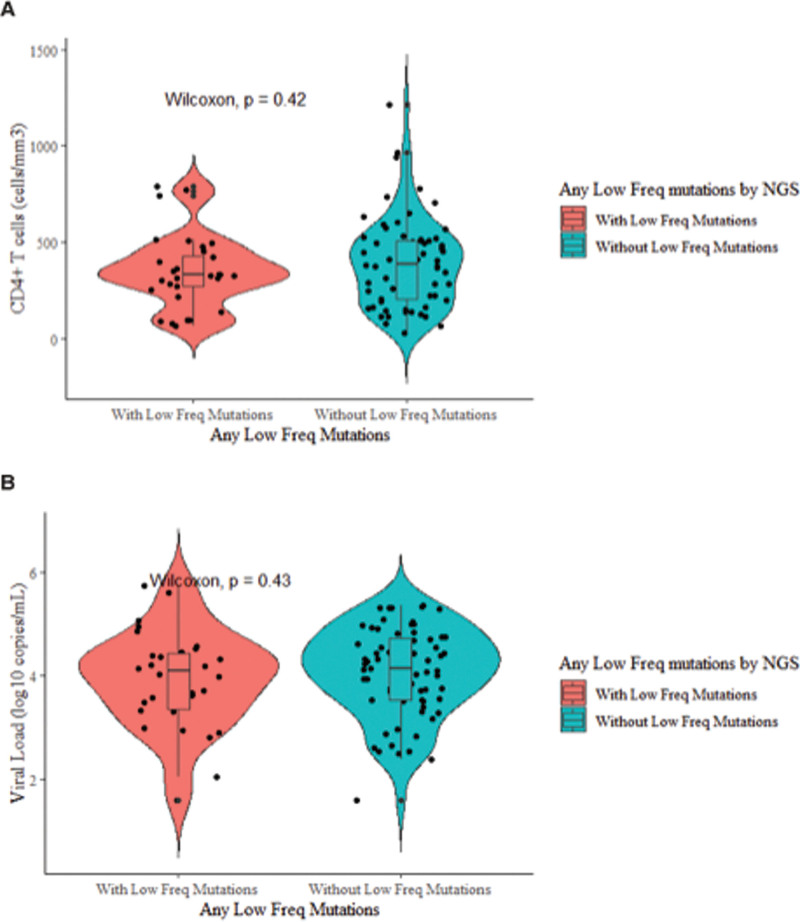
The relationship between the CD4+ T-cell count, viral load and low frequency drug resistance mutations. (A) CD4+ T-cell and (B) viral loads in individuals with low-frequency DRMs and individuals without low-frequency DRMs. Low-frequency DRMs are mutations detected at <20%. DRM, drug resistance mutation. NGS, next-generation sequencing.

The median CD4+ T cell counts for individuals with (n = 33) and without (n = 70) low-frequency DRMs were 331 cell/mm^3^ (IQR, 241–429.5) and 365 cell/mm^3^ (IQR, 227–495), respectively (Fig. 3). In addition, the median VL was 4.2 log_10_ copies/mL (IQR, 3.6–4.6) and 4.1 log_10_ copies/mL (IQR, 3.5–4.6), respectively. There was no difference in the VLs (*P* = .43) and CD4+ T cell counts (*P* = .42) between individuals with low-frequency DRMs and individuals without low-frequency DRMs.

### 3.3. Virological outcome

To determine the impact of low-frequency DRMs, we excluded four individuals with only DRMs detected at ≥20% threshold from further analysis. Seventeen individuals without follow-up VL data were excluded from further analysis. A total of 82 individuals had at least 1 follow-up VL data and were used to investigate the association between baseline low-frequency DRMs and VF (Table 3). Sixty-one of the followed-up participants initiated on ATRIPLA (TDF + FTC + EFV) based regimen while 18 initiated on DTG based regimen (Table S3, Supplemental Digital Content, http://links.lww.com/MD/G927). Twenty-seven of the 82 individuals had low-frequency DRMs, whereas 55 individuals did not. Of the 12 individuals experiencing VF, 4 had low-frequency DRMs. Nine of the 12 individuals initiated an Atripla-based regimen and three individuals initiated on DTG based regimen (Table S3, Supplemental Digital Content, http://links.lww.com/MD/G927). Low-frequency DRMs detected in individuals experiencing VF were NRTI-associated, such as K65R and NNRTI-associated K103N, V108I, and Y188C (Table 4).

**Table 3:** Virological outcome in individuals with and without low-frequency DRMs.

	n	Individuals with low-frequency DRMs (%)	*P*-value
Individuals experiencing VF	12	4 (33.3)	.97
Individuals not experiencing VF	70	23 (32.9)	

DRMs = drug resistance mutations; VF = virological failure.

**Table 4:** DRMs observed in individuals experiencing virological failure with low frequency drug resistance mutations.

Sample identification	Mutation frequency level (%) within the viral	
	NRTI	NNRTI
P26	K65R (2.9%)	0
P29	0	V108I (1.15%)
P51	0	Y188C (2.2%)
P53	0	K103N (7.8%)

NRTI = nucleoside reverse-transcriptase inhibitor; NNRTI = non-Nucleoside reverse transcriptase inhibitor; PI = Protease inhibitor.

Individuals with NNRTI low-frequency DRMs were 2.68 times more likely to experience VF (OR, 2.68; 95% confidential interval, 0.4–13.9) compared with those without NNRTI low-frequency DRM’s.

## 4. Discussion

HIV-1 VF due to the presence or development of HIV variants harboring DRMs remains a major challenge for the success of cART. There are conflicting data on the significance of pretreatment low-frequency HIV DRMs. We report the first study from Botswana that evaluated NGS-defined low-frequency DRMs in antiretroviral naïve individuals. NGS identified all DRMs at levels ≥20% that were detected by Sanger sequencing. The prevalence drastically increased when low-frequency DRMs of 1% were included. Low-frequency DRMs were found in 33 individuals with K65R being the most common low-frequency mutation detected in 8 individuals, and these findings are similar to those reported by others.^[40,50,51]^ The K65R mutation is the most important TDF resistance mutation, which makes it a relevant mutation to the current WHO recommended first-line regimen and may compromise its effectiveness.^[52]^ DRMs detected at levels <20% of viral quasi-species were not detected by Sanger sequencing. The K103N mutation was found in 3 individuals above 5% frequency, and this mutation is known to be highly selected by EFV.^[52]^ The presence of NNRTI DRMs has been suggested to be negatively associated with long-term virologic outcomes of both EFV- and DTG-based first-line ART.^[10]^ Therefore, it is important to continue assessing and monitoring pretreatment mutations in antiretroviral naïve patients. It is also vital to continue assessing NNRTI low-frequency DRMs to ensure optimization of treatment regimens for individuals living with HIV/TB for whom an EFV-based ART may be more appropriate. It has been indicated that there is a difference in CD4 and VL between individuals with low-frequency DRMs and those without low-frequency DRMs.^[50]^ Studies have reported successful amplification and characterization of plasma HIV-1 RNA sequences in patients with VLs below 50 copies/mL.^[53–55]^ A similar finding was also reported in a study conducted in Botswana that used samples with undetectable VLs to determine the prevalence of DRMs.^[56]^ In our study, two samples with VLs of <40 copies/mL were successfully sequenced by Sanger sequencing and NGS. Participants were divided into two groups depending on whether they had low-frequency DRMs. The median VLs did not differ significantly between the group with and without low-frequency DRMs (4.2 log_10_ copies/mL vs 4.1 log_10_ copies/mL), and these findings are consistent with the results reported by Melanie et al.^[57]^

Three of the 4 individuals experiencing VF harbored the most common NNRTI mutations (K103N, V108I, and Y188C), which have been shown to occur more frequently in participants experiencing VF.^[52]^ Individuals with low-frequency NNRTIs associated DRMs were 2.68 times more likely to experience VF (OR, 2.68; 95% confidential interval, 0.4–13.9) compared with those without (*P* = .22) although not statistically significant. This is not unexpected, as it was recently found that the detection of low-frequency DRMs alone was not associated with virologic failure in a South African cohort where HIV-1C also predominates.^[40]^ In a South African study, inclusion of DRMs at >20% to low-frequency DRMs was associated with an increased prediction of VF. In our study, we did not include mutations detected at >20% in our second analysis because genotyping results were communicated in real-time to treat clinicians for appropriate patient management, which might have affected the outcome of our study. It has been previously reported that linked dual-class resistance mutations occurring in a single genome are associated with an increased risk of VF.^[58]^ Since we were not able to analyze viral variants at the single genome level, we could not determine if there was an association of linked dual-class mutations with VF. This study was a retrospective study, and while there were efforts to avoid selection bias, the use of available samples, although it was not intentional. A significant difference was observed in the included versus the excluded in terms of sex and CD4 suggesting that there was some difference between the original cohort and the samples selected for this analysis. Here, we used a threshold of ≥1% frequency, which might have overestimated the prevalence of some mutations, especially K65R; however, the presence of genuine pretreatment DRMs occurring at ≥1% frequency cannot be ruled out.

A limitation of this study was the absence of resistance data at the time of VF. Moreover, we defined VF as a single VL of at least 400 copies/mL, whereas previous studies have used a cutoff of 1000 copies/mL.^[51,59]^ We attempted to amplify samples with low VLs as low as <40 copies/mL, and only 2 samples were successfully amplified. The results should be interpreted with caution, as mutations cannot be reliably detected in such low copies of the VL. Most participants in this study later initiated cART on EFV, a drug that is no longer used in first-line cART therapy in Botswana, so studies investigating the impact of low-frequency DRMS on the current DTG-based first-line regimen are warranted.

In conclusion, the results presented in this study showed that antiretroviral naïve individuals had a high prevalence of low-frequency DRMs that did not have an impact on virologic suppression once they initiated ART. Future studies will need to focus on the role of low-frequency DRMs in DTG-based first-line regimens and to determine the prevalence of low-frequency linked dual-class resistance mutations, which have been found to be more associated with VF.

## Author contributions

Conceptualization: Dorcas Maruapula, Christopher F. Rowley, Melvin Leteane, Sikhulile Moyo, and Simani Gaseitsiwe.

Formal analysis: Dorcas Maruapula, Jennifer Giandhari, Kesaobaka Molebatsi, Olorato Morerinyane, Sikhulile Moyo, and Simani Gaseitsiwe.

Funding acquisition: Dorcas Maruapula, Sikhulile Moyo, and Simani Gaseitsiwe.

Investigation: Dorcas Maruapula, Kaelo K. Seatla, and Jennifer Giandhari.

Methodology: Dorcas Maruapula, Christopher F. Rowley, Jennifer Giandhari, Melvin Leteane, Sikhulile Moyo, and Simani Gaseitsiwe.

Project administration: Dorcas Maruapula.

Resources: Dorcas Maruapula, Christopher F. Rowley, Sikhulile Moyo, Simani Gaseitsiwe.

Supervision: Christopher F. Rowley, Melvin Leteane, Sununguko W. Mpoloka, Sikhulile Moyo, and Simani Gaseitsiwe.

Visualization: Dorcas Maruapula, Sikhulile Moyo, and Simani Gaseitsiwe.

Writing—original draft: Dorcas Maruapula, Christopher F. Rowley, Jennifer Giandhari, Kaelo K. Seatla, Kesaobaka Molebatsi, Olorato Morerinyane, Melvin Leteane, Sununguko W. Mpoloka, Sikhulile Moyo, and Simani Gaseitsiwe.

Writing—review and editing: Dorcas Maruapula, Christopher F. Rowley, Jennifer Giandhari, Kaelo K. Seatla, Kesaobaka Molebatsi, Olorato Morerinyane, Melvin Leteane, Sununguko W. Mpoloka, Rosemary M. Musonda, Tulio de Oliveira, Sikhulile Moyo, and Simani Gaseitsiwe.

## Acknowledgments

The authors would like to acknowledge the study participants, principal investigators, and study coordinators from the novel strategy completed the study. We would also like to extend our acknowledgements to the University of Botswana and the Botswana Harvard HIV Reference Laboratory for their support and contribution to the study’s success. NGS sequence analysis was performed with the assistance of the KwaZulu Natal Research Sequencing Platform and Inqaba biotechnology.

## Supplementary Material


