# Supplementary material for: Low-frequency HIV-1 drug resistance mutations in antiretroviral naïve individuals in Botswana
Source: Medicine (Baltimore). 2022 Jul 15;101(28):e29577. doi: 10.1097/MD.0000000000029577 (PMC11132386; doi:10.1097/MD.0000000000029577)
Supplement: Supplementary file 1 [file medi-101-e29577-s001.pdf]

**Article title:** Low-frequency HIV-1 drug resistance mutations in antiretroviral naïve individuals in Botswana.

**First author:** Dorcas Maruapula

**Table S1.** Comparison of participant characteristics among samples included in analysis and those excluded

| **Characteristics** | **Included individuals (n=108)** | **Excluded individuals (n=423)** | **P-value** |
|---|---|---|---|
| Female, n (%) | 107 (99.1) | 370 (87.5) | <.001 |
| Male, n (%) | 1 (0.9) | 53 (12.5) | <.001 |
| Age in years, median (IQR) | 27 (24-31) | 26 (23-33) | .412 |
| Viral load (log10 copies/mL), median (IQR) | 4.1 (3.5-4.6) | 4.2 (3.5-4.6) | .786 |
| CD4 count (cells/$mm^3$), median (IQR) | 365 (225-497) | 427 (365-514) | <.01 |

IQR = (Interquartile ranges: 25th percentile and 75th percentile); Excluded Participants, refers to participants without available amplicons; Included Participants, refers to participant that had available amplicons and included in final analysis.

**Article title:** Low-frequency HIV-1 drug resistance mutations in antiretroviral naïve individuals in Botswana.

**First author:** Dorcas Maruapula

**Table S2:** Comparison of DRMs identified by Sanger sequencing, PASeq and Hydra

| PID | PASeq | | | Hydra | | | Sanger sequencing | | |
|---|---|---|---|---|---|---|---|---|---|
| | NRTI | NNRTI | PI | NRTI | NNRTI | PI | NRTI | NNRTI | PI |
| P1 | None | Y181C (1.25%) | None | None | None | None | None | None | None |
| P5 | None | None | T74P (1.13%) | None | None | None | None | None | None |
| P7 | None | K103N (98.7%), V106M (1.01%) | None | None | K103N(97.83%) V106I (7.65%) | None | None | K103N | None |
| P8 | M184V, (2.48%) | E138G (1.31%), M230I (2%) | None | M184I(1.66%) | E138G (1.84%), M230I (2.25%) | None | None | None | None |
| P9 | None | None | M46I (1.65%) | None | None | M46I (1.61%) | None | None | None |
| P10 | None | None | | K65R (1.12%) | None | None | None | None | None |
| P11 | None | None | L90M (98.94%) | | None | L90M (99.24%) | None | None | L90M |
| P13 | K65R (1.7%) | None | None | K65R (2.38%) | None | None | None | None | None |
| P14 | K65R (2%) | None | None | K65R (3.97%) | None | None | None | None | None |
| P15 | K65R (1.23%) | None | None | None | None | None | None | None | None |
| P16 | None | None | M46I (13.85%) | None | None | M46I (13.9%) | None | None | None |
| P17 | None | P225H (2%) | I84V (3.03%) | None | P225H (2.46%) | I84V (2.64%) | None | None | None |
| P19 | None | E138G (4.65), V108I (11.25%) | N88S (2.78%) | None | V108I(8.15%) | N88S(4.15%) | None | None | None |
| P21 | None | None | I84V (1.11%) | None | None | | None | None | None |
| P23 | None | None | I50V (1.33%) | None | None | I50V(1.45%) | None | None | None |
| P25 | M41L | Y188H | None | M41L (36.97%) | Y188H | None | M41 | K103N | |

| | | | | | | | | | |
|---|---|---|---|---|---|---|---|---|---|
| | (33.29%) | (1.09%), K103N (97.56%) | | | (1.06%) K103N (98.8%) | | L | | |
| P26 | K65R (2.96%) | None | None | K65R (3.74%) | None | None | None | None | None |
| P27 | K65R (2.18%) | None | M46I (2.08%) | None | None | M46I (2.8%) | None | None | None |
| P28 | A62V (98.99%) | None | None | A62V (99.38%) | None | None | A62V | None | None |
| P29 | Q58E (65.68%) | V108I (1.15%) | None | Q58E (70.49%) | None | None | None | None | Q58E |
| P30 | None | E138K (1.12%) | None | None | None | None | None | None | None |
| P31 | F116Y (2.55%) | V108I (3.55%) | None | K65R (1.65%), F116Y (1.98%) | V108I (1.62%) | None | None | None | None |
| P32 | V75I (2.46%) | None | None | V75I (2.64) | None | None | None | None | None |
| P34 | V75I (5.4%) | None | None | K65R (2.56%), V75I (5.03%) | None | None | None | None | None |
| P36 | None | None | M46L (3.38%) | None | None | M46L (3.23%) | None | None | None |
| P38 | K65R (1%) | None | Q58E (99.71 %) | K65R (1.87) | None | Q58E (99.32%) | None | None | Q58E |
| P41 | None | None | M46I (1.66%) | None | None | M46I (1.19%) | None | None | None |
| P42 | K65R (1.14%) | None | None | K65R (1.63%) | None | None | None | None | None |
| P43 | F77L (4.06%) | None | None | F77L (5.39%) | None | None | None | None | None |
| P44 | M184I (2.43%) | None | None | M184I (2.6%) | None | None | None | None | None |
| P45 | None | E138K (2.74%) | None | None | None | None | None | None | None |
| P46 | None | None | M46I (9.1) | None | None | M46I (8.56%) | None | None | None |
| P47 | K65R (1.23%) | None | | K65R (1.51%) | None | None | None | None | None |
| P49 | None | None | I50V (1.07%) | None | None | None | None | None | None |
| P51 | None | Y188C (2.231%) | None | None | Y188C (1.69%) | None | None | None | None |
| P53 | None | K103N (7.769%) | None | None | K103N (6.93%) | None | None | None | None |
| P56 | None | None | None | K65R (1.32%) | None | None | None | None | None |
| P75 | None | None | None | K65R (1.04%) | None | None | None | None | None |
| P85 | None | K103N(81 .86%) | None | K103N (71.12%) | None | None | None | K103N | None |

| | | | | | | | | | |
|---|---|---|---|---|---|---|---|---|---|
| P94 | M46L (44.49%) | None | None | None | None | M46L (47.43%) | None | None | M46 L |
| P101 | None | None | None | K65R (2.7%) | None | None | None | None | None |

DRMs = drug resistance mutations; NNRTI = non nucleoside reverse transcriptase inhibitor; NRTI = nucleoside reverse transcriptase inhibitor; PI = protease inhibitor; PID = patient identification; PASeq = polymorphism analysis sequencing; None means no drug resistance mutations detected.

**Table S3**: Detailed ART regimens for individuals with follow-up data (n=82)

| PID | Year of ART initiation | Initiation regimen |
|---|---|---|
| P1 | 2014 | ATRIPLA * |
| P3 | 2014 | ATRIPLA* |
| P4 | 2014 | ATRIPLA * |
| P5 | 2015 | ATRIPLA * |
| P6 | 2015 | ATRIPLA* |
| P8 | 2015 | ATRIPLA* |
| P9 | 2016 | ATRIPLA* |
| P10 | 2015 | ATRIPLA |
| P11 | 2015 | ATRIPLA* |
| P12 | 2015 | ATRIPLA* |
| P13 | 2015 | ATRIPLA* |
| P14 | 2015 | ATRIPLA * |
| P16 | 2015 | ATRIPLA * |
| P17 | 2015 | ATRIPLA* |
| P18 | 2016 | DTG + Truvada |
| P19 | 2015 | ATRIPLA* |
| P20 | 2016 | DTG + Truvada |
| P21 | 2015 | ATRIPLA* |
| P22 | 2015 | ATRIPLA |
| P23 | 2015 | ATRIPLA* |
| **P26** | **2017** | **DTG + Truvada** |
| **P29** | **2015** | **ATRIPLA** |
| P30 | 2015 | ATRIPLA* |
| P31 | 2015 | ATRIPLA* |
| P32 | 2012 | ATRIPLA |
| P33 | 2015 | ATRIPLA |
| P34 | 2015 | ATRIPLA |
| P35 | Missing initiation dates | Missing initiation regimen |
| P36 | 2015 | ATRIPLA* |
| P37 | 2015 | ATRIPLA* |
| P39 | 2015 | ATRIPLA |
| P41 | 2015 | ATRIPLA |
| P42 | 2015 | ATRIPLA |
| P46 | 2015 | ATRIPLA* |
| P47 | 2017 | DTG + Truvada |
| P48 | 2015 | ATRIPLA |
| P49 | 2015 | ATRIPLA* |
| P50 | 2015 | ATRIPLA* |
| **P51** | **2016** | **ATRIPLA** |
| P52 | 2016 | ATRIPLA |
| **P53** | **2012** | **ATRIPLA*** |
| P54 | 2018 | DTG +Truvada |
| P55 | 2015 | ATRIPLA |
| P56 | 2017 | DTG + Truvada |
| P57 | 2015 | ATRIPLA |
| P58 | 2015 | ATRIPLA |

| | | |
|---|---|---|
| P59 | 2016 | DTG + Truvada |
| P60 | 2016 | ATRIPLA * |
| P61 | Missing initiation dates | Missing initiation regimen |
| P62 | 2015 | ATRIPLA* |
| **P63** | **2015** | **ATRIPLA*** |
| P64 | 2016 | DTG + Truvada |
| **P65** | **2016** | **ATRIPLA*** |
| P68 | 2016 | ATRIPLA* |
| P69 | 2015 | ATRIPLA* |
| P70 | 2015 | ATRIPLA |
| P71 | 2016 | DTG + Truvada * |
| P73 | 2016 | DTG + Truvada * |
| P74 | 2012 | ATRIPLA * |
| P75 | 2016 | ATRIPLA |
| **P76** | **2012** | **ATRIPLA*** |
| P77 | 2017 | DTG + Truvada |
| **P78** | **2013** | **ATRIPLA** |
| P79 | 2016 | DTG + Truvada * |
| **P80** | **2016** | **DTG + Truvada** |
| **P81** | **2017** | **DTG +Truvada *** |
| **P82** | **2012** | **ATRIPLA** |
| **P83** | **2014** | **ATRIPLA** |
| P84 | 2014 | ATRIPLA * |
| P86 | 2018 | DTG +Truvada |
| P87 | 2015 | ATRIPLA |
| P89 | 2018 | ATRIPLA |
| P91 | 2016 | DTG + Truvada * |
| P93 | 2015 | ATRIPLA |
| P95 | 2015 | ATRIPLA * |
| P97 | Missing initiation dates | Missing initiation regimen |
| P98 | 2015 | ATRIPLA |
| P99 | 2017 | DTG + Truvada |
| P100 | 2015 | ATRIPLA * |
| P101 | 2016 | DTG + Truvada * |
| P102 | 2015 | ATRIPLA |
| P103 | 2015 | ATRIPLA |

PID = Patient identification; ATRIPLA = TDF+FTC+EFV; DTG = dolutegravir; Truvada = TDF+FTC; * Inferred initiation regimen based on initiation dates. Bold = Individuals who experienced virological failure (n=12).
